# Clinical Significance and Risk Factors of Local Recurrence in Synovial Sarcoma: A Retrospective Analysis of 171 Cases

**DOI:** 10.3389/fsurg.2021.736146

**Published:** 2022-01-13

**Authors:** Hongqiang Zhang, Wending Huang, Qi Feng, Wei Sun, Wangjun Yan, Chunmeng Wang, Jianing Zhang, Kai Huang, Lin Yu, Xinglong Qu, Yong Chen

**Affiliations:** ^1^Department of Surgical Oncology, Shanghai Cancer Center Minhang Branch Hospital, Fudan University, Shanghai, China; ^2^Department of Musculoskeletal Oncology, Shanghai Cancer Center, Fudan University, Shanghai, China; ^3^Department of Oncology, Shanghai Medical College of Fudan University, Shanghai, China; ^4^Department of Orthopedics, The Fourth Hospital of Hebei Medical University, Shijiazhuang, China; ^5^Department of Surgery Base, Shanghai Cancer Center, Fudan University, Shanghai, China; ^6^Department of Surgery, Brandon Regional Hospital, Morsani College of Medicine, Hospital Corporation of America Healthcare/University of South Florida Health, Tampa, FL, United States; ^7^Department of Pathology, Shanghai Cancer Center, Fudan University, Shanghai, China

**Keywords:** synovial sarcoma, local recurrence, overall survival (OS), prognosis, risk factor

## Abstract

**Objective:** To investigate risk factors of local recurrence of synovial sarcoma and the impact of local recurrence on survival.

**Methods:** We retrospectively reviewed clinical data of patients with II to IIIB (AJCC8) synovial sarcoma who underwent surgery at our center between March 2005 and December 2016. Data relating clinicopathological factors, treatment and prognosis were collected. The impact of local recurrence on overall survival (OS), local recurrence-free survival (LRFS), and distant relapse-free survival (DRFS) were analyzed. The prognostic factors associated with local recurrence were also analyzed using Kaplan-Meier Curves and Cox regression analysis.

**Results:** A total of 171 patients were included in this analysis. After a median follow-up of 48 months, 66 patients (38.6%) experienced local recurrence. The 5-year OS, LRFS, and DRFS rates of patients with local recurrence were 37.6, 6.1, and 24.1%, respectively. Multivariate analysis showed that larger initial tumors, multiple recurrences, positive resection margins, marginal resection, and lack of adjuvant therapy were associated with higher local recurrence.

**Conclusion:** Local recurrence of synovial sarcoma is associated with distant metastasis and poor survival. Chemoradiation improves the prognosis of patients with local recurrence, in particular those for which recurrence occurs shortly after initial treatment.

## Introduction

Synovial sarcoma (SS) is a malignant tumor that accounts for 5–10% of soft tissue sarcomas (STS) (1) and is most common in teenagers and young adults. Histologically, SS can be divided into three subtypes: monophasic fibrous, biphasic, and poorly differentiated SS. Recent studies assessing molecular genetics have shown that over 90% of SS cases are characterized by the t (X; 18) (p11.2; q11.2) translocation, which results in the formation of an oncogenic fusion gene (SS18-SSX1, SS18-SSX2, or SS18-SSX4) (2–4). The standard treatment for SS is complete removal of the tumor with a negative resection margin and peri-operative radiation if indicated. However, the role of adjuvant chemotherapy remains controversial and has only been retrospectively confirmed to improve survival in high-risk patients (5). Previous clinical studies have demonstrated that prognostic factors for SS survival include age (6–8), initial tumor size (6, 8, 9), local recurrence (LR) (9, 10), surgical method of resection (11), histological subtype (8, 12), gene fusion type (13, 14), metastasis (9, 13–17), and post-operative radiotherapy (18). Recently, it has been reported that LR may affect prognosis in STS patients (19, 20), but there is limited evidence on the impact of LR on prognosis of SS patients. The purpose of this study was to investigate the impact of LR on the prognosis of SS and to explore the risk factors associated with LR.

## Materials and Methods

We retrospectively analyzed the clinical data of patients with SS who underwent surgery at our center between March 2005 and December 2016. All patients underwent standardized computed tomography (CT) or magnetic resonance imaging (MRI) and chest CT scans to assess local and distant metastasis (DM). Pre-operative percutaneous biopsies or incision biopsies were performed in newly diagnosed patients. The diagnosis was confirmed by histopathology and immunohistochemistry for all 171 participants included in this study. In 146 (146/171, 85.4%) patients, SS18-SSX chromosome fusion gene testing was carried out for further confirmation of diagnosis. The exclusion criteria included as follows: (1) DM or tumors other than SS at the initial visit; (2) follow up <3 years, or missing follow-up data. Data on clinical characteristics, treatment, and outcome were analyzed as part of this study.

All patients underwent surgery, which included: radical resection, wide resection, and marginal resection. Post-operative radiotherapy was performed for high-risk patients (i.e. patients with an initial tumor size ≥5 cm, deep location of the tumor (in relation to the fascia), positive resection margins, or patients who exhibited recurrence). The target area was the tumor bed and the surrounding edges (2–3 cm). The median total radiation dose was 60 Gy, ranging between 50 and 75 Gy. Adjuvant chemotherapy was carried out with either MAID (Mesna + Adriamycin + Ifosfamide + Dacarbazine) or AIM (Adriamycin + Ifosfamide + Mesna) in high-risk patients who received 4 to 6 courses of adjuvant chemotherapy treatment. Reoperation was considered for patients with recurrence or limited metastasis, if radical resection was possible. Patients with unresectable or diffuse metastatic disease were offered palliative treatment including surgery, chemoradiotherapy, targeted therapies, interventional ablation, or hyperthermic perfusion chemotherapy. For advanced SS patients that were not previously exposed to anthracyclines, anthracycline-based chemotherapy was the standard treatment regimen. Conversely, second-line treatments such as a GVP regimen (Gemcitabine + Vincristine + Cisplatin) were used in palliative chemotherapy (21).

Patients were followed up every 3 months for the first 2 years after surgery, then twice a year between years 2 and 3 after surgery, and subsequently once a year. LR and DM were determined by objective clinical, radiological, or histological examination. The primary outcome was OS, LRFS, and DRFS. Statistical analysis was performed using SPSS 25.0 software. Standard Kaplan-Meier analysis was used to develop the survival curve and estimate OS, LRFS, and DRFS. Univariate analysis of prognosis was performed. A Cox proportional hazards regression model was used to analyze factors affecting prognosis. All statistical tests were two-sided and a *p* < 0.05 was considered statistically significant.

## Results

### Clinical Characteristics, Pathological Features, and Treatment Variables

A total of 171 patients with SS were included in this retrospective analysis. Clinical characteristics of included patients are summarized in Table 1. Seventy-two (72/171, 42.1%) patients were readmitted to hospital due to recurrence, of whom 16 (16/72, 22.2%) relapsed twice or more, and 30 (30/72, 41.7%) exhibited early recurrence (i.e., within 12 months of the original disease). Tumors were predominantly located in lower limbs (94/171, 55.0%) with a median size of 5 cm (1–23 cm). Most tumors were located deep below the fascia. T staging was carried out based on the maximum diameter of tumors at different locations (AJCC8).

**Table 1:** Clinical, pathological, and therapeutic information of 66 cases with local recurrence and 105 cases without recurrence after surgery at our center.

**Variable**	**Total**	**Recurrence**	**No recurrence**	***P***
	**No**.	**No. (%)**	**No. (%)**	
Total	171	66 (38.6)	105 (61.4)	
**Gender**				0.009
Male	79	38 (48.1)	41 (51.9)	
Female	92	28 (30.4)	64 (69.6)	
**Age, y**				0.862
Median	36	36	37	
Range	14–80	16–80	14–78	
≤ 30 y	68	27 (39.7)	41 (60.3)	
>30 y	103	39 (37.9)	64 (62.1)	
**History of recurrence**				<0.001
No	99	24 (24.2)	75 (75.8)	
Primary disease	25	6 (24.0)	18 (76.0)	
After incomplete excision	74	18 (24.3)	55 (75.7)	
One	56	28 (50.0)	28 (50.0)	
Two or more	16	14 (87.5)	2 (12.5)	
**Recurrence interval** ^**a**^				0.010
Median	15 months	8 months	27 months	
Range	2–90 months	2–11 months	13–90 months	
Early recurrence	30	22 (73.3)	8 (26.7)	
Late recurrence	42	20 (47.6)	22 (52.4)	
**Number of recurrences** ^**b**^				/
Zero	74	0	74 (100.0)	/
One	47	18 (38.3)	29 (61.7)	/
Two or more	50	48 (96.0)	2 (4.0)	/
**Size (AJCC8)**				<0.001
Median	5 cm	7 cm	4 cm	
Range	0.8–23 cm	1.2–23 cm	0.8–16 cm	
T1	96	21 (21.9)	75 (78.1)	
T2	44	23 (52.3)	21 (47.7)	
T3	18	11 (61.1)	7 (38.9)	
T4	13	11 (84.6)	2 (15.4)	
**Position**				<0.001
Upper limbs	20	4 (20.0)	16 (80.0)	
Lower limbs	94	33 (35.1)	61 (64.9)	
Head and neck	9	4 (44.4)	5 (55.6)	
Trunk	41	18 (43.9)	23 (56.1)	
Internal organ	7	7 (100)	0	
**Depth**	0.009			
Superficial	21	3 (14.3)	18 (85.7)	
Deep	150	63 (42.0)	87 (58.0)	
**Invasion of adjoining structures**				0.301
Yes	50	16 (32.0)	34 (68.0)	
No	121	50(41.3)	71 (58.7)	
**Histologic subtype**				0.164
Monophasic fibrous	92	30(32.6)	62 (67.4)	
Biphasic	48	21 (43.8)	27 (56.2)	
Poorly differentiated	31	15 (48.4)	16 (51.6)	
**Stage (AJCC8)**				<0.001
II	96	21 (21.9)	75 (78.1)	
III	75	45 (60.0)	30 (40.0)	
**Type of surgery**				<0.001
Radical resection	55	10 (18.2)	47 (81.8)	
Amputation	15	2 (13.3)	13 (86.7)	
Joint replacement	8	1 (12.5)	7 (87.5)	
Complete muscle group resection	32	7 (21.9)	25 (78.1)	
Wide resection	80	31 (38.8)	49 (61.2)	
Marginal resection	36	25 (69.4)	11 (30.6)	
**Resection margins (UICC)**				<0.001
R0	139	42 (30.2)	97 (69.8)	
R1/2	32	24 (75.0)	8 (25.0)	
**Post-operative radiotherapy**				0.007
Yes	86	28 (32.6)	59 (67.4)	
No	85	38 (44.7)	45 (55.3)	
**Adjuvant chemotherapy**				0.047
Yes	75	24 (32.0)	51 (68.0)	
No	96	42 (43.8)	54 (56.2)	

*AJCC, American Joint Committee on Cancer; UICC, Union for International Cancer Control.*

*^a^Recurrence interval in 72 patients with a history of recurrence; ^b^Number of recurrences up to the last follow-up. /, This factor was not analyzed*.

Types of surgery and resection margins are listed in Table 1. Amputations or joint replacements were performed in 16 patients with recurrent disease (16/72, 22.2%), compared to 7 patients without recurrent disease (7/99, 7.1 %). Vascular reconstruction was performed in 8 patients with major vascular involvement. In patients with tumors abutting or encasing important nerves, dissection around the nerve was performed and anhydrous alcohol was used as an adjunct during surgery. Twenty-seven (27/171, 15.8%) patients had R1 resections, while 5 (5/171, 2.9%) cases had R2 resections. In 5 patients with marginal resection, tumor rupture occurred during surgery as a result of inappropriate handling; these were considered as having had R2 resections. Post-operative radiotherapy was performed in 86 (86/171, 50.3%) patients. Eight (8/171, 4.7%) patients received 2 to 4 cycles of neoadjuvant chemotherapy before surgery, and 75 (75/171, 43.9%) patients received adjuvant chemotherapy. Of the 66 patients who relapsed after surgery, 27 (27/66, 40.9%) patients were repeatedly treated with surgery (12 of these also received adjuvant chemotherapy), 25 (25/66, 37.9%) patients received palliative chemotherapy only, 2 (2/66, 3.0%) patients received perfusion chemotherapy only, and treatment was aborted in the remaining 13 (13/66, 19.7%) patients.

### Impact of Local Recurrence on OS

**Figure 1:**
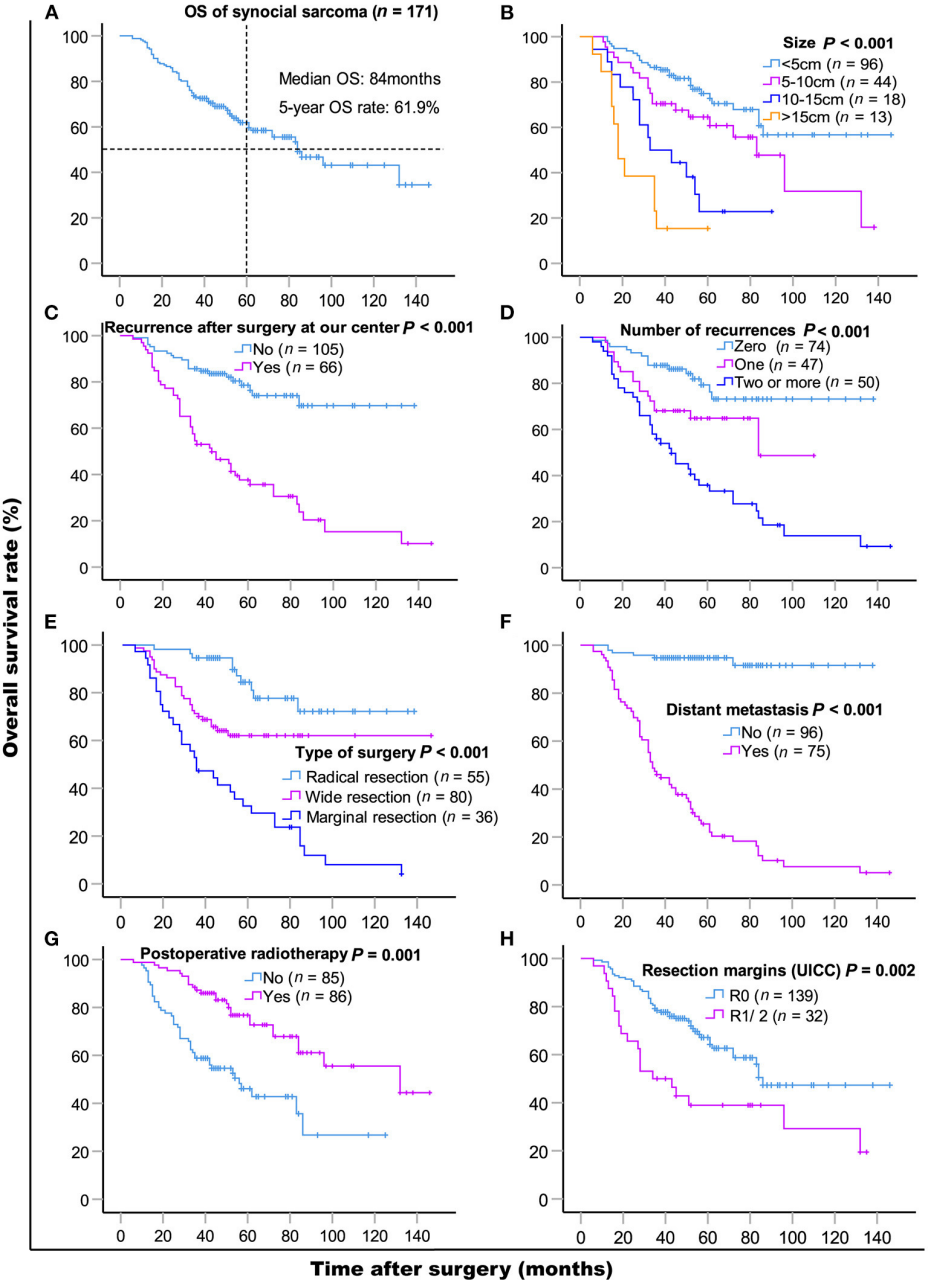
Kaplan-Meier overall survival curves of patients with synovial sarcoma **(A)** and compared according to **(B)** tumor size, **(C)** recurrence after surgery at our center, **(D)** the number of recurrences, **(E)** type of surgery, **(F)** distant metastasis, **(G)** post-operative radiotherapy, **(H)** resection margins (UICC). Log-rank test was used to compare curves, number of patients in subgroup (*n*) and significance (*P*) are shown on panels.

The median follow-up time was 48 months (range 5–143 months). As of the last follow-up, 71 (71/171, 41.5%) patients died of SS. The median OS was 84 months, and the 5- and 10-year OS rates were 61.9% [95% CI, 54.06–69.74] and 43.1% (95% CI, 30.95–55.25), respectively. Results of the univariate and multivariate OS analyses are shown in Table 2. Cox multivariate analysis revealed that initial tumor sizes ≥5 cm, LR, DM, marginal resection, and non-post-operative radiotherapy were independent predictors of worse survival. The 5-year OS rate was 37.6% in 66 patients with LR, and 78.6% in 105 patients without recurrence (*P* < 0.001), respectively. As of the last follow-up, 74 (74/171, 43.3%) patients did not exhibit LR, 47 (47/171, 27.5%) patients had one recurrence, and 50 (50/171, 29.2%) patients had two or more recurrences. The 5-year OS rates were 79.3%, 64.8%, and 35.8% (*P* < 0.001), respectively (Figure 1). The median recurrence interval was 20 months (*n* = 66 patients), with the median OS of the early and late recurrence groups being 18 and 61 months, respectively (*P* < 0.001). Among the 66 patients with LR, 27 (27/66, 40.9%) were repeatedly treated with surgery and had a median survival of 54 months after recurrence. The 5-year OS rates of the patients with or without DM were 94.7 and 25.4%, respectively (*P* < 0.001). In patients with advanced metastatic SS, the median survival times of patients for whom treatment was abandoned those who received palliative chemotherapy only were 6 and 10 months, respectively (*P* = 0.036).

**Table 2:** Univariate and multivariate analysis of variable factors and OS.

**Factors**	**5-year OS**	**10-year OS**	**Univariate**		**Multivariate**	
			**HR (95% CI)**	***P***	**HR (95%CI)**	***P***
**Gender**						
Male	55.3	29.2	1.829 (1.140–2.933)	0.011	1.530 (0.907–2.580)	0.111
Female	67.4	56.4	1.000	Reference	1.000	Reference
**History of recurrence**				0.001		0.104
No	71.6	56.9	1.000	Reference	1.000	Reference
One	52.3	NA	1.957 (1.172–3.269)	0.010	1.585 (0.755–3.328)	0.223
Two or more	34.3	11.4	3.184 (1.630–6.219)	0.001	1.813 (1.036–3.171)	0.037
**Recurrence interval** ^**a**^	/	/				
Early recurrence	30.8	NA	1.876 (0.992–3.548)	0.048	/	/
Late recurrence	60.8	31.8	1.000	Reference	/	/
**Recurrence** ^**b**^						
No	78.6	69.7	1.000	Reference	1.000	Reference
Yes	37.6	15.3	4.040 (2.454–6.649)	<0.001	1.967 (1.002–3.864)	0.049
**Distant metastasis**						
No	94.7	91.6	1.000	Reference	1.000	Reference
Yes	25.4	7.6	22.404 (9.681–51.843)	<0.001	19.584(8.150–47.063)	<0.001
**Number of recurrences** ^**c**^	<0.001		0.347			
Zero	79.3	73.2	1.000	Reference	1.000	Reference
One	64.8	NA	2.143 (1.069–4.297)	0.032	1.344 (0.547–3.302)	0.520
Two or more	35.8	13.9	4.735 (2.606–8.601)	<0.001	2.173 (0.706–6.692)	0.176
**Size (AJCC8)**				<0.001		0.002
T1	74.9	56.7	1.000	Reference	1.000	Reference
T2	64.6	31.8	1.790 (1.003–3.196)	0.049	1.481 (0.803–2.731)	0.209
T3	22.9	NA	3.983 (2.040–7.778)	<0.001	1.980 (0.993–3.949)	0.053
T4	15.4	NA	8.431 (4.063–17.494)	<0.001	4.480 (2.078–9.659)	<0.001
**Depth**						
Superficial	92.3	63.5	1.000	Reference	1.000	Reference
Deep	57.5	39.8	3.438 (1.250–9.458)	0.010	1.497 (0.503–4.449)	0.468
**Stage (AJCC8)**						
II	74.5	53.7	1.000	Reference	1.000	Reference
III	45.2	26.1	2.543 (1.580–4.092)	<0.001	3.811 (0.450–32.296)	0.220
**Type of surgery**				<0.001		0.001
Radical resection	84.4	72.1	1.000	Reference	1.000	Reference
Wide resection	62.0	62.0	2.849 (1.382–5.873)	0.005	3.119 (1.484–6.555)	0.003
Marginal resection	32.5	7.9	7.165 (3.516–14.603)	<0.001	4.232 (1.9381–9.245)	<0.001
**Resection margins (UICC)**						
R0	67.1	47.3	1.000	Reference	1.000	Reference
R1/2	39.0	29.2	2.235 (1.334–3.745)	0.002	1.856 (0.879–3.921)	0.105
**Post-operative radiotherapy**						
Yes	76.8	55.5	1.000	Reference	1.000	Reference
No	46.1	26.8	2.745 (1.670–4.513)	0.001	3.290 (1.905–5.682)	<0.001
**Adjuvant chemotherapy**						
Yes	70.5	53.4	1.000	Reference	1.000	Reference
No	54.4	33.4	1.653(1.011–2.702)	0.042	1.048 (0.570–1.924)	0.881

*OS, overall survival; HR, hazard ratio; CI, confidence interval; AJCC, American Joint Committee on Cancer; UICC, Union for International Cancer Control; NA, Not Available.*

*^a^Recurrence interval in 72 patients with a history of recurrence; ^b^Recurrence after surgery at our center; ^c^Number of recurrences up to the last follow-up; /, There were not enough numbers to conduct a multivariate analysis*.

### Impact of Local Recurrence on DRFS

A total of 75 (75/171, 43.9%) patients had DM, of which 69 (69/75, 92.0%) had lung metastasis (9 patients exhibited multiple DM), and 6 (6/75, 8.0%) only had regional lymph node metastasis. The median DRFS was 84 months (1–139 months). The 5- and 10-year DRFS were 54.1 and 44.2%, respectively. The results of the univariate and multivariate DRFS analyses are shown in Table 3. Multiple recurrences (*P* <0.001, HR: 3.161, 95% CI: 1.674–5.969) were the most important prognostic factor associated with metastasis in both univariate and multivariate analysis. The rate of metastasis stratified by recurrence was as follows: 23.0% (17/74) for patients that did not exhibit recurrence, 36.2% (17/47) for patients with a single recurrence, and 82% (41/50) for patients two or more recurrences.

**Table 3:** Univariate and multivariate analysis of variable factors and DRFS.

**Factors**	**5-year DRFS**	**Univariate**		**Multivariate**	
		**HR (95% CI)**	***P***	**HR (95% CI)**	***P***
**History of recurrence**			<0.001		0.001
No	66.9	1.000	Reference	1.000	Reference
One	46.4	2.051 (1.246–3.379)	0.005	2.425 (1.420–4.143)	0.007
Two or more	8.80	3.708 (1.941–7.084)	<0.001	2.828 (1.323–6.002)	0.001
**Recurrence** ^**a**^					
No	73.9	1.000	Reference	1.000	Reference
Yes	24.1	4.338 (2.686–7.005)	<0.001	1.974 (1.104–3.529)	0.022
**Number of recurrences** ^**b**^			<0.001		<0.001
Zero	74.1	1.000	Reference	1.000	Reference
One	64.5	1.752 (0.894–3.434)	0.102	1.403 (0.705–2.792)	0.335
Two or more	17.7	5.660 (3.202–10.003)	<0.001	3.161 (1.674–5.969)	<0.001
**Size (AJCC8)**			<0.001		0.001
T1	68.2	1.000	Reference	1.000	Reference
T2	48.4	1.926 (1.106–3.355)	0.021	1.121 (0.619–2.027)	0.707
T3	16.7	5.127 (2.731–9.624)	<0.001	2.748 (1.199–6.300)	0.017
T4	28.8	5.314 (2.482–11.376)	<0.001	3.980 (1.953–8.108)	<0.001
**Depth**					
Superficial	74.6	1.000	Reference	1.000	Reference
Deep	51.2	3.437 (1.253–9.432)	0.010	2.245 (0.784–6.424)	0.132
**Stage (AJCC8)**					
II	66.0	1.000	Reference	1.000	Reference
III	37.9	2.637 (1.662–4.184)	<0.001	4.680 (0.851–6.663)	0.076
**Type of surgery**			0.001		<0.001
Radical resection	71.0	1.000	Reference	1.000	Reference
Wide resection	58.7	1.893 (1.019–3.514)	0.043	2.335 (1.198–4.549)	0.013
Marginal resection	20.6	5.142(2.743–9.640)	<0.001	4.817 (2.321–9.997)	<0.001
**Resection margins (UICC)**					
R0	57.8	1.000	Reference	1.000	Reference
R1/2	36.9	2.285 (1.377–3.795)	0.001	1.322 (0.709–2.467)	0.380
**Post-operative radiotherapy**					
Yes	62.4	1.000	Reference	1.000	Reference
No	44.9	1.711 (1.080–2.711)	0.020	2.159 (1.270–3.670)	0.004
**Adjuvant chemotherapy**					
Yes	61.3	1.000	Reference	/	/
No	47.9	1.305 (0.820–2.076)	0.256	/	/

*DRFS, distant recurrence-free survival; HR, hazard ratio; CI, confidence interval; AJCC, American Joint Committee on Cancer; UICC, Union for International Cancer.*

*^a^Recurrence after surgery at our center; ^b^Number of recurrences up to the last follow-up; /, There was no statistical significance in univariate analysis*.

### Impact of Local Recurrence on LRFS and Risk Factors of LRFS

**Figure 2:**
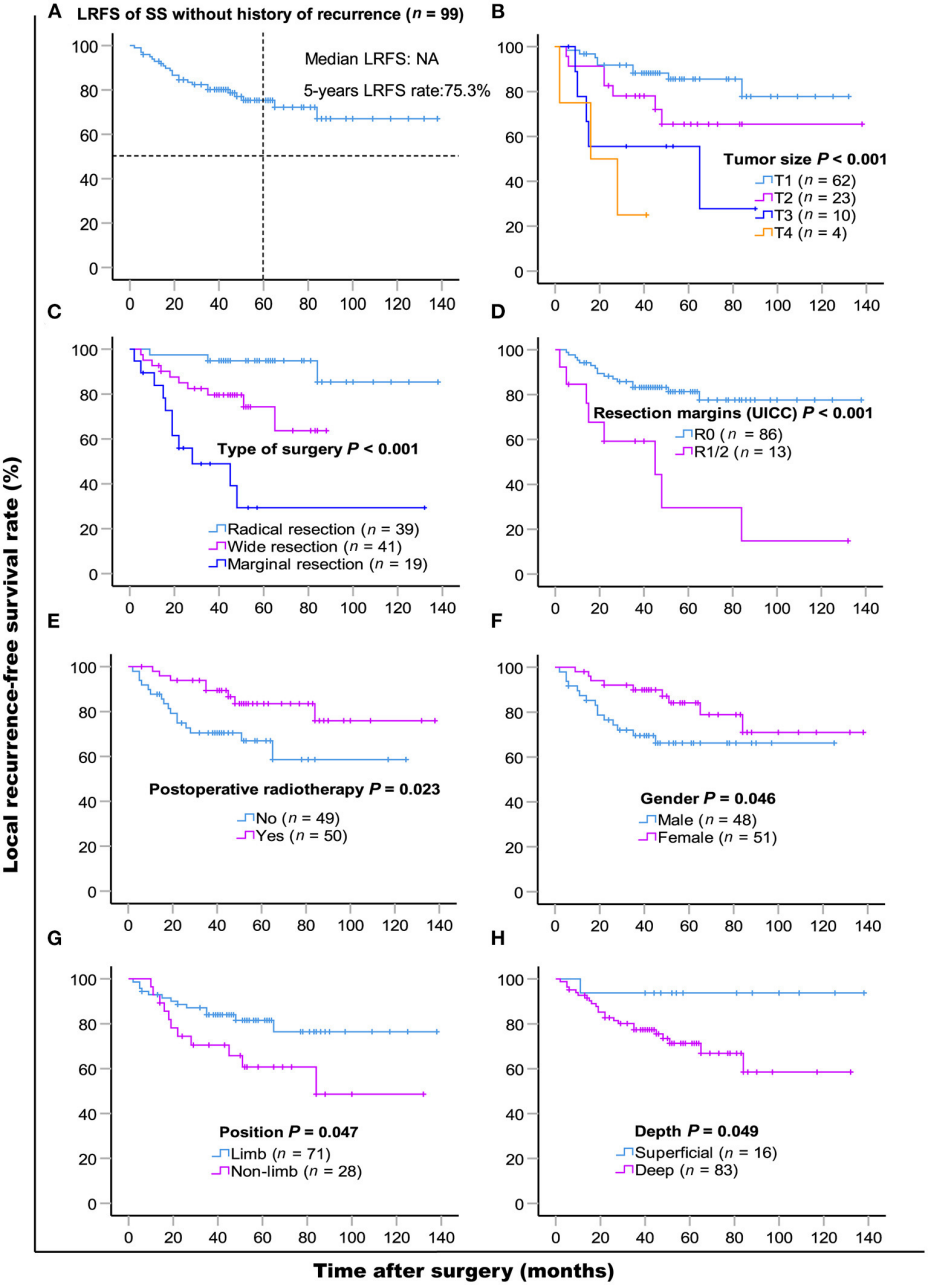
Kaplan-Meier local recurrence-free survival curves of 99 patients without history of recurrence **(A)** and compared according to **(B)** tumor size, **(C)** type of surgery, **(D)** resection margins (UICC), **(E)** post-operative radiotherapy, **(F)** gender, **(G)** position, **(H)** depth. Log-rank test was used to compare curves, number of patients in subgroup (*n*) and significance (*P*) are shown on panels.

LR occurred in 38.6% (66/171) of patients. The 5- and 10- year LRFS were 59.6% (95% CI, 51.56–67.64) and 51.3% (95% CI, 40.72–61.88), respectively. The recurrence rate was 58.3% (42/72) in patients with a prior history of recurrence. The 5-year LRFS rates of patients with history of zero, one, and two or more recurrences were 75.3, 46.5, and 10.4%, respectively (*P* < 0.001). The median LRFS of patients with early and late recurrence were 31 and 48 months (*P* = 0.010, HR: 0.465 95% CI, 0.255–0.849), respectively. We analyzed risk factors for LR in 99 patients without a history of recurrence disease. The results are shown in Table 4 and Figure 2. Univariate analysis showed that the following prognostic factors were associated with a high risk of LR (*P* < 0.05): male sex, large and deep tumors, non-limb sites, positive resection margins, late-stage tumors, marginal resection, and a lack of post-operative radiotherapy. Multivariate analysis showed that marginal resection (*P* < 0.001, HR: 3.370, 95% CI: 3.370–50.809), large tumors (*P* = 0.004, HR: 5.338, 95% CI: 1.723–16.533), late stage tumors (*P* = 0.019, HR: 7.223, 95% CI: 1.386–37.653), lack of post-operative radiotherapy (*P* = 0.028, HR: 2.945, 95% CI: 1.121–7.735), and positive resection margins (*P* = 0.032, HR: 4.307, 95% CI: 1.135–16.344) were significant independent poor-prognostic factors associated with LR.

**Table 4:** LRFS in 99 patients with synovial sarcoma without history of recurrence, as determined with univariate and cox multivariate analyses.

**Factors**	**No**.	**5-year LRFS**	**Univariate**		**Multivariate**	
			**HR (95% CI)**	***P***	**HR (95% CI)**	***P***
**Gender**						
Male	48	66.2	2.278 (0.992–5.229)	0.046	2.414 (0.985–5.915)	0.054
Female	51	84.1	1.000	Reference	1.000	Reference
**Position**						
Limb	71	76.4	1.000	Reference	1.000	Reference
Non-limb	28	60.7	2.209 (0.988–4.940)	0.047	1.916490 (0.577–3.848)	0.410
**Size (AJCC8)**				<0.001		0.010
T1	62	85.6	1.000	Reference	1.000	Reference
T2	23	65.5	2.329 (0.865–6.267)	0.094	1.625 (0.595–4.439)	0.344
T3	10	55.6	5.135 (1.713–15.387)	0.003	5.189 (1.252–21.497)	0.023
T4	4	NA	10.851 (2.850–41.315)	<0.001	5.338 (1.723–16.533)	0.004
**Depth**						
Superficial	16	93.8	1.000	Reference	1.000	Reference
Deep	83	71.3	5.936 (0.795–44.302)	0.049	3.793 (0.456–31.578)	0.218
**Stage (AJCC8)**						
II	62	85.6	1.000	Reference	1.000	Reference
III	37	58.6	3.503 (1.528–8.027)	0.002	7.223 (1.386–37.653)	0.019
**Type of surgery**				<0.001		0.001
Radical resection	39	94.9	1.000	Reference	1.000	Reference
Wide resection	41	74.3	4.319 (1.178–15.834)	0.027	4.018 (1.077–14.991)	0.038
Marginal resection	19	29.4	14.572 (3.974–53.432)	<0.001	13.086 (3.370–50.809)	<0.001
**Resection margins (UICC)**						
R0	86	81.3	1.000	Reference	1.000	Reference
R1/2	13	29.6	4.751 (2.021–11.172)	<0.001	4.307 (1.135–16.344)	0.032
**Post-operative radiotherapy**						
Yes	50	83.5	1.000	Reference	1.000	Reference
No	49	67.0	2.600 (1.105–6.118)	0.023	2.945 (1.121–7.735)	0.028
**Adjuvant chemotherapy**						
Yes	41	81.9	1.000	Reference	/	/
No	58	70.4	2.025 (0.836–4.907)	0.110	/	/

*LRFS, local recurrence-free survival; HR, hazard ratio; CI, confidence interval; AJCC, American Joint Committee on Cancer; UICC, Union for International Cancer; NA, Not Available.*

*/, There was no statistical significance in univariate analysis*.

## Discussion

SS is a high-grade soft tissue sarcoma with high rates of recurrence and metastasis. The negative effect of LR on long-term survival of patients has been previously demonstrated. For instance, Bergh et al. (10) showed that LR results in a 3.6-fold increase in mortality. In our study, we found an 1.967-fold increase in risk of death of patients with recurrence compared to those without. Although the number of recurrences was negatively correlated with OS in univariate analysis, it was no longer a significant prognostic factor in multivariate analysis. However, the number of SS recurrences was a risk factor for LR and DM, which were in turn important factors influencing survival time.

In our study, 79.1% of recurrences and 73.0% of metastasis occurred during the first 3 years of initial diagnosis, and we found a significant correlation between LR and DM. Consistent with the research by Deshumukh (9), more than half of patients with a history of recurrence experienced another relapse, and 82.0% of the patients with multiple relapses developed metastasis. Importantly, the interval at which recurrence occurred following initial diagnosis was significantly correlated with OS and LR. Patients with early recurrence after the initial surgery (i.e. within the first 12 months) had a higher risk of recurrence (HR: 2.148) and earlier relapses, but it may be related to almost half of these patients received unplanned resection. Early recurrence usually indicates a high risk for metastasis. In our study, we identified that larger initial tumor sizes, a previous history of recurrence, marginal resection, and positive resection margins are risk factors for early LR. We found that both early and multiple recurrences are indicators of a poorer prognosis.

A history of multiple recurrences is associated with a higher risk of LR. In order to assess risk factors other than a prior history of recurrence, we next analyzed the risk factors of LR in patients without a history of recurrence. Multivariate analysis revealed that larger and late-stage tumors significantly increased the risk of LR. Radical resection with negative margins was a key determinant of reducing the risk of LR. Post-operative radiotherapy reduced the risk of recurrence by 0.660 (HR: 0.340). Male patients were more likely to relapse than female patients. Interestingly, this may be related to a frequency of the SYT-SSX1 fusion transcript, which has been shown to be independently associated with an increased risk of early recurrence (22), in males.

Given the poor prognosis of recurrent SS, it is difficult to treat via localized surgery and more aggressive resection is often required. Therefore, initial treatment is critical for the prognosis of SS, impacting patient mortality and recurrence rates. At present, surgical resection with negative margins in combination with radiotherapy is widely used in patients with SS. Repeat surgery has an overall positive effect for the management of recurrent SS. The NCCN guidelines recommend radiotherapy as a standard adjuvant therapy after surgery (23). A large study previously found that perioperative radiotherapy in SS patients was associated with higher negative margin rates and better outcomes (24). In line with this, we also found that radiotherapy was beneficial for local tumor control. Pisters et al. (25) reported a 5–10% LR rate for T1 primary sarcomas with microscopically positive (R1) final surgical margins, after using of radiation. O'Donnell et al. (26) showed that the LR rate was 15% for positive margins in the setting of radiotherapy. Although more than half of the patients included in this study received post-operative radiotherapy, LR rates of T1 and T4 tumors were 21.9 and 84.6%, respectively. This was likely due to the high proportion of patients with a prior history of recurrence in our study. In the 99 patients without a history of recurrence, the LR rate of T1 tumors was 14.5%.

The role of chemotherapy in the prognosis of SS is still controversial, although SS is generally considered a chemosensitive disease (17). A synovial sarcoma-specific study demonstrated a survival benefit for patients treated with ifosfamide-based chemotherapy pre-operatively (22). Phase III randomized clinical trials conducted by Gronchi et al. (27) confirmed that neoadjuvant chemotherapy with high-dose AI can improve the prognosis of high-risk STS patients. However, Italiano et al. (28) found that neoadjuvant chemotherapy and/or adjuvant chemotherapy had no significant effect on OS, LRFS, and DMFS. In our study, we found that chemotherapy is an important adjuvant option for high-risk SS (29). In this study, we also demonstrated that adjuvant chemotherapy can improve OS in SS patients. Here, we showed that palliative chemotherapy improved the survival of patients with advanced SS, consistent with the result of a previous study (5). However, the number of patients undergoing neoadjuvant chemoradiotherapy was too small to conduct a full statistical analysis, requiring further future research.

## Conclusion

Local recurrence of SS is a key risk factor for OS, early and multiple recurrences are indicators of a poorer prognosis. The identification of prognostic factors for LR is required to obtain better control and guide comprehensive treatment of patients in order to achieve better survival rates. According to our study, early detection of tumors, early radical resection with negative margins, and multidisciplinary comprehensive treatments can help reduce the LR and therefore improve the prognosis of SS.

## Data Availability Statement

The original contributions presented in the study are included in the article/supplementary material, further inquiries can be directed to the corresponding author/s.

## Ethics Statement

The studies involving human participants were reviewed and approved by the Institutional Review Board of Fudan University Shanghai Cancer Center approved this study (1310128-1 & 1310128-1-1312). The patients/participants provided their written informed consent to participate in this study.

## Author Contributions

HZ: formal analysis, investigation, visualization, writing—original draft, and writing—review and editing. WH and QF: formal analysis, investigation, supervision, visualization, writing—original draft, and writing—review and editing. WS and JZ: investigation and writing—review and editing. KH: writing—review and editing. WY and CW: formal analysis, methodology, and writing—review and editing. LY: investigation, methodology, and writing—review & editing. XQ: conceptualization, investigation, resources, supervision, and writing—review and editing. YC: conceptualization, funding acquisition, investigation, resources, supervision, and writing—review and editing. All authors contributed to the article and approved the submitted version.

## Funding

This work was supported by the grants from the Beijing Medical and Health Foundation (YWJKJJHKYJJ-F2189E).

## Conflict of Interest

The authors declare that the research was conducted in the absence of any commercial or financial relationships that could be construed as a potential conflict of interest.

## Publisher's Note

All claims expressed in this article are solely those of the authors and do not necessarily represent those of their affiliated organizations, or those of the publisher, the editors and the reviewers. Any product that may be evaluated in this article, or claim that may be made by its manufacturer, is not guaranteed or endorsed by the publisher.

## Abbreviations

OS: overall survival
LRFS: local recurrence-free survival
DRFS: distant relapse-free survival
SS: synovial sarcoma
STS: soft tissue sarcomas
LR: local recurrence
CT: computed tomography
MRI: magnetic resonance imaging
DM: distant metastasis
MAID: Mesna + Adriamycin + Ifosfamide + Dacarbazine
AIM: Adriamycin + Ifosfamide + Mesna
GVP: Gemcitabine + Vincristine + Cisplatin
AJCC: American Joint Committee on Cancer.
